# Characterization of the complete mitochondrial genome and phylogenetic relationship of *Neodon sikimensis* (Rodentia: Arvicolinae)

**DOI:** 10.1080/23802359.2016.1180561

**Published:** 2016-07-08

**Authors:** Qi Zhang, Tingting He, Haixue Wei, Fengjun Li, Yuanchao Feng, Hao Zong, Shunde Chen

**Affiliations:** College of Life Sciences, Sichuan Normal University, Chengdu, China

**Keywords:** Arvicolinae, complete mitochondrial genome, phylogenetic relationship, Sikkim mountain vole

## Abstract

The Sikkim Mountain vole (*Neodon sikimensis*) belongs to the subfamily Arvicolinae. In this study, we sequenced the complete mitochondrial genome of *N. sikimensis*. It was determined to be 16,330 bp long and contained 13 protein-coding genes, 2 ribosomal RNA genes, 22 transfer RNA genes and 1 control region. The nucleotide sequence data of 12 heavy-strand protein-coding genes of *N. sikimensis* and other 22 rodents were used for phylogenetic analysis. Tree constructed using Bayesian phylogenetic methods demonstrated that *N. sikimensis* was a sister to *N. irene*, and *Microtus genus* species did not cluster together with each other, making the genus a paraphyletic group.

The Rodentia has more than 2200 species in the word (Wilson & Reeder 2005). In China, there are more than 210 species (Jiang et al. 2015). The genus *Neodon* Horsfield 1841 is classified within the subfamily Arvicolinae (Musser & Carleton 2005), most of which lack comprehensive biological data. The Sikkim Mountain vole (*Neodon sikimensis*) belongs to the subfamily Arvicolinae, which is distributed in southern Tibet, Nepal, northeastern India and Bhutan (Smith et al. 2009). This species is restricted to alpine meadows and dense vegetation growing at the edges of rhododendron and coniferous forest, at the elevations of 2100–3700 m (Smith et al. 2009). It prefers to feed on green vegetation or seeds. In this study, we sequenced the complete mitochondrial genome of *N. sikimensis* (16, 330 bp; GenBank accession no. KU891252), to provide reference information for further study on Arvicolinae species.

This individual was captured in Motuo County, Tibet (latitude: 29°28′0.3″N, longitude: 94°59′59.3″E). A voucher specimen was deposited at the College of Life Sciences, Sichuan Normal University. The whole mitochondrial genome of *N. sikimensis* contains a set of 13 protein-coding genes, two ribosomal RNA genes (rRNA), 22 transfer RNA genes (tRNA) and 1 control region. The gene order and gene content of the mitochondrial genome of *N. sikimensis* is identical to that observed in most other Cricetidae species (Fan et al. 2011; Yang et al. 2012; Chen et al. 2015).

In order to explore the molecular phylogenetics evolution of Arvicolinae, the nucleotide sequence data of 12 heavy-strand protein-coding genes of *N. sikimensis* and other 22 rodents were used for the phylogenetic analysis. After alignment, the sequence set contained 10,788 bp. We used BEAST v1.7.4 (Drummond & Rambaut 2007) for Bayesian phylogenetic reconstructions for mitochondrial genomes of other 22 rodents. The best-fit GTR + I + G model of DNA substitution was obtained using jModelTest v2 (Darriba et al. 2012) under the Akaike Information Criterion (AIC). *Sicista concolor* was used as outgroup.

Tree constructed using Bayesian phylogenetic analysis is shown in Figure 1. The 22 ingroup species sampled in this study belonged to two families: Muridae and Cricetidae. *Neodon sikimensis* clustered with species *Neodon irene*, *Lasiopodomys mandarinus*, *Microtus fortis* and *Microtus kikuchii*, formed a solid monophyletic group, well supported by a Bayesian posterior probability of 1.00 (Figure 1). Bayesian analyses suggested that *N. sikimensis* was a sister to *N. irene* (Bayesian posterior probability =1.00). Furthermore, the BI tree demonstrated that *Microtus levis*, *Microtus ochrogaster*, *Microtus fortis* and *Microtus kikuchii* did not cluster together with each other. In order to have better understanding in the phylogenetic relationship within Arvicolinae, more complete mitochondrial genome sequences are required. We expect the study contributes to species identification and may facilitate further investigation of the molecular evolution of Arvicolinae.

**Figure 1.**
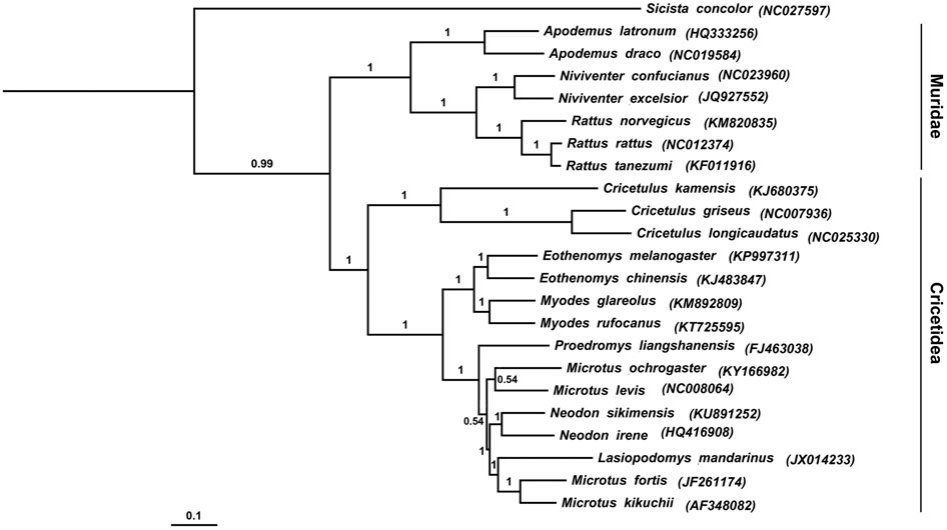
Bayesian phylogenetic analyses for *Neodon sikimensis* based on complete mitochondrial genome.
